# Intersociety policy statement on the use of whole-exome sequencing in the critically ill newborn infant

**DOI:** 10.1186/s13052-017-0418-0

**Published:** 2017-11-03

**Authors:** Alessandro Borghesi, Maria Antonietta Mencarelli, Luigi Memo, Giovanni Battista Ferrero, Andrea Bartuli, Maurizio Genuardi, Mauro Stronati, Alberto Villani, Alessandra Renieri, Giovanni Corsello, on behalf of their respective Scientific Societies, Rino Agostiniani, Annamaria Staiano, Antonio Del Vecchio, Giuseppe Banderali, Diego Peroni, Ruggiero Piazzolla, Renato Turra, Elena Bozzola, Davide Vecchio, Giovanni Vitali Rosati, Francesco Macrì, Costantino Romagnoli, Mario De Curtis, Luigi Orfeo, Giovanna Mangili, Fabio Mosca, Vassilios Fanos, Piermichele Paolillo, Francesco Raimondi, Angelo Rizzo, Gaetano Chirico, Luigi Tommaso Corvaglia, Caterina Cacace, Carlo Poggiani, Antonio Alberto Zuppa, Denis Pisano, Daniela Melis, Mario Giuffrè, Maria Teresa Carbone, Marina Macchiaiolo, Luigi Tarani, Angelo Selicorni, Generoso Andria, Domenico Coviello, Mattia Gentile, Paola Ghiorzo, Antonio Novelli, Angela Ragusa, Gioacchino Scarano, Daniela Giardino

**Affiliations:** 1 Neonatal Intensive Care Unit, Fondazione IRCCS Policlinco San Matteo, Piazzale Golgi, 19, 27100 Pavia, Italy; 2 Genetica Medica, Azienda Ospedaliera Universitaria Senese, Siena, Italy; 3 Pediatric Department, S. Martino Hospital, Belluno, Italy; 4 Department of Public Health and Pediatrics, University of Torino, Torino, Italy; 5 Rare Diseases and Medical Genetic Unit, Bambino Gesù Children’s Hospital, IRCCS, Rome, Italy; 6 Institute of Genomic Medicine, Università Cattolica Del Sacro Cuore, Fondazione Policlinico A. Gemelli, Rome, Italy; 7 Pediatric and Infectious Disease Unit, Bambino Gesù Children’s Hospital, IRCCS, Rome, Italy; 8 Medical Genetics, University of Siena, Siena, Italy; 9 Operative Unit of Pediatrics and Neonatal Intensive Therapy, Mother and Child Department, University of Palermo, Palermo, Italy

**Keywords:** Neonate, Genome, Whole-exome sequencing, WES, WGS, Genetic, Mendelian, NICU, Neonatal intensive care unit, Diagnosis

## Abstract

The rapid advancement of next-generation sequencing (NGS) technology and the decrease in costs for whole-exome sequencing (WES) and whole-genome sequening (WGS), has prompted its clinical application in several fields of medicine. Currently, there are no specific guidelines for the use of NGS in the field of neonatal medicine and in the diagnosis of genetic diseases in critically ill newborn infants. As a consequence, NGS may be underused with reduced diagnostic success rate, or overused, with increased costs for the healthcare system. Most genetic diseases may be already expressed during the neonatal age, but their identification may be complicated by nonspecific presentation, especially in the setting of critical clinical conditions. The differential diagnosis process in the neonatal intensive care unit (NICU) may be time-consuming, uncomfortable for the patient due to repeated sampling, and ineffective in reaching a molecular diagnosis during NICU stay. Serial gene sequencing (Sanger sequencing) may be successful only for conditions for which the clinical phenotype strongly suggests a diagnostic hypothesis and for genetically homogeneous diseases. Newborn screenings with Guthrie cards, which vary from country to country, are designed to only test for a few dozen genetic diseases out of the more than 6000 diseases for which a genetic characterization is available. The use of WES in selected cases in the NICU may overcome these issues. We present an intersociety document that aims to define the best indications for the use of WES in different clinical scenarios in the NICU. We propose that WES is used in the NICU for critically ill newborn infants when an early diagnosis is desirable to guide the clinical management during NICU stay, when a strong hypothesis cannot be formulated based on the clinical phenotype or the disease is genetically heterogeneous, and when specific non-genetic laboratory tests are not available. The use of WES may reduce the time for diagnosis in infants during NICU stay and may eventually result in cost-effectiveness.

## Background

Genetic disorders, including single-gene disorders, copy number variations (CNVs) and chromosomal abnormalities, are individually rare conditions, yet overall affecting a substantial proportion of the general population [1, 2]. To date, a molecular characterization is available for 6011 of the more than 7000 identified genetic diseases [3].

The field of medical genetics has gained an increasingly important role in recent years. The development of novel techniques to investigate the genetic bases of human phenotypes and the concurrent reduction in the costs for genomic analyses has boosted scientists and clinicians to include the use of genomic techniques in clinical practice [4].

The application of next-generation sequencing (NGS) technology to interrogate the genomes of individuals, families and cohorts in order to identify the molecular defects responsible for diseases is maximally important in the field of neonatal medicine. Indeed, despite the fact that genetic disorders may occur as late-onset conditions (onset in adolescence or adulthood), many genetic conditions are already clinically expressed during the first 28 days of life or shortly after [5].

Specific guidelines or recommendations for the use of whole-exome sequencing (WES) and whole-genome sequencing (WGS) in the critically ill neonate are lacking. The aim of the present intersociety policy statement is to provide a rationale for the use of WES in the critically ill newborn infant, to identify possible clinical scenarios, and to set out the best indications for different techniques in each scenario.

### Epidemiology of genetic disorders in the neonatal intensive care unit (NICU)

The actual incidence of genetic diseases in the NICU is unknown because many potentially genetic conditions are often not appropriately investigated during hospital stay, or genetic tests are performed later on in life. Moreover, some genetic conditions may be silent or only partially expressed and not easily recognizable during the neonatal age.

A major group of diseases occurring in neonatal units with potential genetic origin are congenital anomalies, also referred to as birth defects or congenital malformations. Birth defects occur in 2–3% of live births [6–9], congenital heart disease in 1% [10], and inborn errors of metabolism in 0.5% [11]. It has been estimated that birth defects, neuromuscular disorders, neurodevelopmental delay and intellectual disability overall affect 10% of all live births.

Approximately 20% of infant mortality in the US is estimated to occur due to birth defects and/or chromosomal abnormalities [12]. Despite the reduction by 50% of the overall neonatal mortality thanks to the improvement in neonatal intensive care techniques, mortality due to birth defects has remained stable across the past 30 years. Both genetic and non-genetic etiologies have been identified, including prenatal infections, parental exposure to teratogens, events occurring during pregnancy and, among genetic factors, chromosomal aneuploidy, copy number variations and monogenic conditions.

Monogenic disorders, also referred to as single-gene disorders, are diseases due to genetic defects disrupting the function of one single gene. The resulting phenotype may be highly variable in terms of organ and tissue involvement, clinical severity and age at onset. Currently, 4969 monogenic disorders have been described, caused by genetic variations in 3378 genes [3]. The actual incidence of monogenic disorders underlying clinical disease in the NICU is unknown.

### Importance of the etiologic diagnosis in the NICU

The molecular characterization of a disease phenotype in the NICU may have fundamental practical implications. First, it can provide a diagnostic response to families. Second, if specific treatments are available for the identified disease, it provides the rationale for the use of targeted therapies and withdrawal of empirical, ineffective or potentially harmful treatments, thus resulting in life-saving changes in the clinical management of the patient. Further, parents can be provided with detailed prognostic counseling useful to predict potential late-onset complications, to design preventive strategies and rehabilitation, and to early acces specific therapeutic protocols or programs for infants with special needs. Moreover, the molecular characterization of the patient’s disease may help parents to better plan their future reproductive choices based on the risk of familial recurrence, and to early identify the disease genes in other current or future family members [5]. Finally, the etiologic definition of the clinical phenotype may be useful, in critical care settings for diseases with unfavourable prognosis, to discuss with the family the most appropriate end-of-life decisions. Altogether, targeted interventions in the critical newborn based on the molecular etiology of the disease may result in improved clinical management and in overall reduced mortality and morbidity in the NICU and in the involved families.

### Technologies for clinical genetic testing in the NICU

A comprehensive description of all available techniques for genetic testing is beyond the scope of the present review. Here we briefly list the techniques that are more commonly used for the diagnosis of genetic disorders in clinical settings, and further focus on the clinical application of NGS methodologies.

The identification of neonatal diseases through the non-genetic testing with the Guthrie cards (newborn screening) is highly sensitive and rapid, can efficiently detect actionable neonatal disorders, and is an invaluable prevention tool, but it is designed to test only a limited number of genetic diseases [13–16].

Microarray techniques, including array-Comparative Genomic Hybridization (array-CGH) and single nucleotide polymorphism-array (SNP-array), are the techniques of choice for the identification of chromosomal aneuploides and CNVs [17]. The standard karyotype is necessary for the diagnosis of balanced chromosomal abnormalities (e.g. balanced translocations) that are usually not detected with microarrays. Because cranio-facial defects, multiple birth defects, neurodevelopmental delay, intellectual disability and pervasive developmental disorders are often caused by chromosomal abnormalities or CNVs, array-CGH or SNP-array are the first-line technologies for the characterization of these disorders [18].

Serial gene sequencing (e.g. Sanger sequencing) can be successfully used, in the setting of suspected monogenic conditions, to study a limited number of genetic defects. However, serial gene sequencing is often too slow to provide useful information in the short term during NICU stay, especially for genetically heterogeneous diseases (defects in several genes underlying the same phenotype), and a diagnosis is often returned after patient’s discharge or death. Furthermore, it requires a strong diagnostic hypothesis and prior knowledge of the disease-causing genes. As a consequence, the clinical management of the newborn infant in the NICU seldom relies on genetic information obtained through serial gene sequencing.

The NGS technology is based on the so-called massive parallel sequencing and can be used to sequence the whole genome of an individual (i.e., WGS), or specific target regions, in few days. The most widely used application of NGS is WES [19], consisting of the simultaneous sequencing of all the coding regions of the genome (the exons). NGS is designed to identify single nucleotide variations (SNVs) and small insertions/deletions (InDels), and is primarily used for the diagnosis of monogenic disorders.

WES and WGS have also been applied to the analysis of CNVs [20–22], but array-CGH is currently the first-line technology to use when diseases caused by CNVs are suspected.

### Clinical applications of NGS

Since the first publication in 2009 on a genetic diagnosis using the NGS technology [23], a series of publications on both pediatric and adult patients have unquestionably demonstrated the clinical utility of WES/WGS [24]. In adult and pediatric patients, NGS, and specifically WES, has been demonstrated to be able to change the decision-making processes at the bedside, to guide the long-term clinical management, and to affect the prognosis, when actionable conditions are identified [25].

Similarly, NGS has been successfully applied to obtain the molecular explanation of complex fetal and neonatal conditions with nonspecific or incomplete syndromic presentations or for genetically heterogeneous conditions. In some cases, the molecular characterization of the condition allowed to modify the clinical management and the therapeutic decisions [26–28].

For birth defects, the diagnostic success rate of WES is 20–25% when the analysis is restricted to known disease-genes, and may be as high as 45% for specific conditions as, for instance, neurodevelopmental disorders [2]. As a comparison, the diagnostic success rate is ~25% for Sanger sequencing and ~20% for microarray techniques and standard karyotype [29].

Specific study designs and diagnostic strategies may increase the success rate of NGS.

Family- or trio-based analyses are designed to test specific and the most likely genetic hypotheses (autosomal dominant, autosomal recessive, X-linked) or to restrict the analysis to variants shared by affected and absent in unaffected members of the kindred [30].

The success rate of NGS increases when the patient’s history and description, including laboratory and imaging studies, are reported in detail and with high accuracy. WES associated with the so-called deep clinical phenotyping achieved a success rate of 68% for the molecular characterization of inborn errors of metabolism, with actionable diagnoses obtained in 44% of all cases [25].

Reaching a genetic diagnosis through WES or WGS is complex and laborious. The huge amount of data generated during a NGS experiment must be handled by expert bioinformaticians. Several potentially pathogenic variants of unknown significance are identified during a single WES/WGS experiment and data interpretation is a critical step. The identification of the causative variant responsible for the phenotype under study, when achieved, is based on hard filtering or variant prioritization according to several criteria [31]. These include allele frequency of the identified genetic variant in public databases (e.g., gnomAD, 1 kg) [32, 33], predicted loss-of-function or pathogenicity of the variant, the most likely genetic model (autosomal dominant, autosomal recessive, X-linked, de novo), the conservation of the protein domain, gene function, and previous publications reporting proof of causality between the same variant or other variants in the same gene and the patient’s phenotype.

Data interpretation has greater success when performed as a multidisciplinary process. The first fundamental step is the accurate phenotyping of the patient and the annotation of all the relevant clinical, laboratory and instrumental signs by the neonatologist or by the clinical geneticist, which allows a more effective variant prioritization based on gene function or on previous associations between variants and similar phenotypes in public databases (e.g. ClinVar) [34].

### Use of NGS for the molecular diagnosis in the newborn infant

Difficulties in the molecular diagnosis of neonatal conditions include:

- Phenotypic variability (incomplete penetrance, variable expressivity);
- Genetic heterogeneity, even for highly homogenous and clinically recognizable phenotypes, complicating the diagnostic workup;
- Poor index of suspicion for a genetic condition in the neonatal age because i) the disease phenotype is not fully expressed early in life, or ii) clinical signs are subtle and highly aspecific or masked, especially in the setting of critical conditions.

These infants usually undergo extensive testing with repeated blood sampling and imaging studies, that are often unable to provide a molecular characterization or a diagnostic response.

NGS technology allows the simultaneous sequencing of more than 20,000 genes (for WES) or a panel of preselected genes, and can be efficiently used in clinical settings to at least partially overcome these issues. For this reason, beyond applications in research projects, an increasing number of clinical laboratories is using NGS-based genetic testing for the diagnosis of suspected genetic conditions.

Because a substantial proportion of known genetic conditions may have clinical impact during the first 28 days of life, the NICU is among the medical environments with highest potential for successful application of NGS technology.

For neonatal medicine, NGS technology may have a critical role for the diagnosis of:

i) Suspected genetic conditions which are phenotypically nonspecific and not consistent with any known syndrome;
ii) A condition consistent with a genetic disease known to be genetically heterogenous, for which non-genetic laboratory testing is not available, and for which classical Sanger sequencing would be costly and would require a long diagnostic time;
iii) Suspected genetic conditions in the critically ill neonate for which a rapid diagnosis is relevant to the clinical decision-making process in the NICU.

Moreover, a timely diagnosis during hospital stay may result in a shorter time for the differential diagnosis, shorter NICU stay, use of specific treatments and avoidance of nonspecific and ineffective drugs and, ultimately, in an overall reduction of the healthcare costs for the patient. Therefore, with the progressive reduction in the costs for NGS, a timely diagnosis with NGS in selected NICU patients may eventually result in cost-effectiveness [5, 27].

### Current advantages of WES over WGS

WES is designed to only identify genetic variations in the exonic regions and the flanking intronic regions (1–1.5% of the whole genome), while most genomic non-coding regions are not covered by the analysis. Nevertheless, WES is the most widespread application of NGS to identify rare genetic variations underlying Mendelian disorders because of its cost-effectiveness. Indeed, approximately 85% of the identified human disease-causing variants are located in the exonic regions [35, 36].

Better coverage and slightly higher sensitivity in detecting genetic variants, especially in splice-site and intronic regions flanking the exons, may be obtained with WGS [37]. However, despite a reduction in the costs over the past few years, WGS is still less cost-effective than WES, and requires a longer time for analysis and higher computational costs [38]. In the future, a further reduction in the costs for sequencing and the rapid evolution of the technology together with the development of novel protocols for rapid diagnostic WGS may decrease the computational time and resources needed for WGS and tip the balance towards the use of WGS as first-line test for clinical use [5, 27].

### Policy statement on the use of WES in the NICU

The above considerations represent the rationale for defining the diagnostic indications to the use of WES in the critically ill newborn infant.

The boards of the four involved scientific societies designated one or two members from each society to draft together the first version of the policy statement. The draft circulated among all the members of the boards of each involved scientific society. Each board member edited the draft and expressed his/her concerns. A final version in Italian language, addressing all the concerns and accepting the proposed modifications, was eventually drafted, approved by the members of the boards, and published on the websites of the four involved scientific societies.

**Fig. 1:**
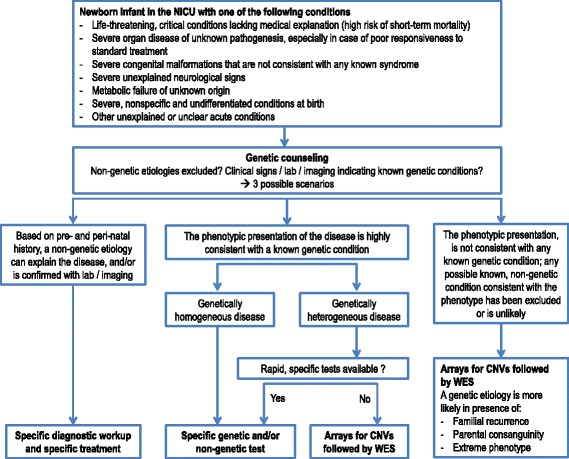
Diagnostic algorithm for genetic testing in the critically ill newborn infant

As a general indication, the current intersociety policy statement proposes that WES should be performed in critically ill newborn infants if a diagnostic hypothesis is not achievable based on the sole clinical phenotype, and for suspected conditions with a highly heterogenous molecular pathogenesis (Fig. 1).

Specifically, a genetic diagnosis should always be seeked in the following situations:

i) Life-threatening, critical conditions (e.g. cardiopulmonary failure, multiorgan failure) lacking medical explanation (high risk of short-term mortality);
ii) Severe organ disease of unknown pathogenesis, especially in case of poor responsiveness to standard treatment;
iii) Severe congenital malformations that are not consistent with any known syndrome;
iv) Severe unexplained neurological signs;
v) Metabolic failure of unknown origin;
vi) Severe, nonspecific and undifferentiated conditions at birth;
vii) Other unexplained acute conditions;

The above listed conditions can occur in three possible scenarios:

**Scenario 1**. A non-genetic etiology is the most likely explanation for the clinical conditions based on the pre- and peri-natal history, and/or has been confirmed with laboratory and imaging studies. The diagnostic workup for the specific disease should be followed. The patient should be treated according to the specific therapeutic protocol.

**Scenario 2**. The phenotype is highly consistent with a known genetic condition:

- If the disease is known to be genetically homogeneous (few genes are known to underlie the disease), if serial gene sequencing can provide results in a short time, and/or if non-genetic, rapid laboratory tests (e.g. biochemical) are readily available, the patient should be tested for defects in selected genes or undergo specific laboratory testing;

- If the disease is genetically heterogeneous, if rapid non-genetic laboratory tests are not available, and a genetic laboratory with experience in the disease cannot provide a diagnostic response with serial gene sequencing in a short time, the patient should undergo array-based genetic testing for CNVs, followed by WES in case of negative results.

**Scenario 3**. The phenotype, regardless of the complexity of the clinical picture, is not consistent with any known genetic condition, and any possible known, non-genetic condition consistent with the phenotype has been excluded. In such a scenario, the patient should undergo array-based genetic testing for CNVs followed by WES in case of negative results.

The presence of familial recurrence or consanguineous parents in the patient’s history, a severe form of a commonly mild phenotype (the so-called “extreme phenotype”), and the slow or absent reponse to drugs that are usually effective, increase the likelihood of (although their absence does not exclude) a genetic condition.

Genetic counseling should always be requested prior to genetic analyses to obtain guidance on the opportunity to perform genetic testing, to obtain information on the most appropriate genetic diagnostic workup, or before excluding the utility of any genetic analyses in the specific clinical case.

Genetic counseling with the families should also be planned after the results of the genetic tests are available to provide prognostic information to the parents and design the most appropriate health program for the patient and the family.

The importance of establishing an early genetic diagnosis in these conditions cannot be overstated. A genetic diagnosis has critical practical implications, including avoidance of unnecessary and repeated blood sampling and invasive diagnostic studies, administration of patient-targeted treatments, establishment of disease-specific, protocol-based follow-up programs, prevention of potential long-term complications and sequelae often associated with a diagnostic delay of a genetic disease, and, in case of unfavourable prognosis, discussion with the family about the most appropriate end-of-life decisions.

If a fulminant and fatal form of a disease does not allow the reaching of a diagnostic response before patient’s death, biological samples from the patient should always be stored for subsequent diagnosis, which may be useful to the family to predict the risk of familial recurrence.

### Ethical considerations

General ethical issues related to clinical use of WES include: i) potential identification of genetic defects that are unrelated to the clinical phenotype for which WES was indicated, sometimes associated with a late-onset phenotype or a carrier status; ii) potential identification of variants of unknown significance; iii) privacy and data security issues, particularly sensitive for individuals in the pediatric age.

The decision on which secondary data should be communicated is a matter of debate [39]. In some countries there is agreement on which incidental findings can be communicated [39]. The American College of Medical Genetics and Genomics published and recently updated a minimum list of genes to be reported as incidental or secondary findings, with the aim of preventing or significantly reducing morbidity and mortality for selected highly penetrant genetic disorders [40]. Specifically for newborn infants, the “BabySeq project” recently published a curated gene list for reporting results of newborn genomic sequencing [41].

The list of genes to report is linked to the current knowledge, and the rapid discovery of novel variants and novel disease-genes will require periodic update of the lists of genetic variations and genes to be communicated.

The current policy of the Italian Society of Human Genetics (Società Italiana di Genetica Umana, SIGU) is to rely on the patient’s or parent’s decision, after detailed information has been provided and informed consent has been signed [42]. A possible solution is to decide prior to the analysis which data should be communicated. In the most restrictive option, only variants related to the disease for which WES was indicated that are actionable for the patient or other family members will be communicated while variants of unknown origin or variants predisposing to late-onset diseases or associated with carrier status, that do not modify the short term clinical decisions, will not be communicated.

The Institutional Review Board of the institution should always be involved when specific situations do not allow an agreement on a specific communication policy with the parents.

Regarding privacy and security issues, specific investments from political and healthcare institutions and organizations are required to guarantee safe long-term storage of the genomic data after appropriate de-linking of the patient’s name with the stored data.

### Potential future applications of NGS in neonatal medicine

#### Newborn screening

The use of NGS for newborn screening is object of discussion [43–45]. Before NGS can be integrated in the universal newborn screening, however, several issues should be solved, including ethical (e.g. communication of results to families, ownership of the data), financial (costs for sequencing and data storage), security (appropriate encryption/decryption methods) and technical issues (time for sequencing and bioinformatics analysis, interpretation of the results, optimization of the starting material for DNA extraction) [46].

#### Use of rapid WGS for genetic diagnosis in the NICU

The use of rapid WGS for targeted genetic diagnosis in the NICU was first suggested by a research group at Children’s Mercy Hospital (Kansas City, MO, USA), and WGS alone or in combination with RNA-sequencing is currently used by other groups [5, 27]. By reducing the sequencing time (rapid mode sequencing) and automatizing the bioinformatics pipeline, researchers have been able to provide a genetic diagnosis in NICU, from blood sampling to results, in 26 h, with a success rate of 57% when applied to a carefully selected population of patients [38]. Despite being promising in clinical trials, similar results would not be easily obtained in daily clinical practice. Indeed, the widespread adoption of rapid WGS or WGS + RNA-sequencing is nowadays costly and requires highly specific expertise. WES currently remains the methodology of choice in laboratories for the diagnosis of monogenic conditions in the NICU.

## Conclusions

Specific recommendation or guidelines for the use of NGS technology in the neonatal age are lacking. Most genetic diseases are clinically relevant during the neonatal age. The diagnosis of genetic diseases with serial gene sequencing or with non-genetic testing may be time-consuming, uncomfortable for the patient, and often ineffective in reaching a molecular diagnosis during NICU stay. The present intersociety document proposes to use WES for genetic diagnosis in critically ill newborn infants when a strong hypothesis on the likely genetic diseases cannot be reached based on the phenotype, a rapid diagnosis may lead to changes in clinical management during hospital stay, or when the suspected genetic condition is genetically heterogeneous. The use of WES may reduce the time for diagnosis in infants during NICU stay while being cost-effective.

## Abbreviations

Array-CGH: Array-comparative genomic hybridization
CNV: Copy number variation
NGS: Next-generation sequencing
NICU: Neonatal intensive care unit
SNP-array: single nucleotide polymorphism-array
WES: Whole-exome sequencing
WGS: Whole-genome sequencing
